# Development of a questionnaire specifically for patients with Ileal Orthotopic Neobladder (IONB)

**DOI:** 10.1186/s12955-014-0135-y

**Published:** 2014-09-01

**Authors:** Salvatore Siracusano, Mauro Niero, Cristina Lonardi, Maria Angela Cerruto, Stefano Ciciliato, Laura Toffoli, Francesco Visalli, Davide Massidda, Massimo Iafrate, Walter Artibani, Pierfrancesco Bassi, Ciro Imbimbo, Marco Racioppi, Renato Talamini, Carolina D’Elia, Giovanni Cacciamani, Davide De Marchi, Tommaso Silvestri, Paolo Verze, Emanuele Belgrano

**Affiliations:** Department of Urology, Trieste University, Cattinara Hospital Via Strada di Fiume 447, 34100 Trieste, Italy; TESIS Department, Verona University, Verona, Italy; Department of Urology, Verona University, Verona, Italy; Department of Urology, Padua University, Padua, Italy; Department of Urology, Università Cattolica Policlinico Gemelli Roma, Gemelli Roma, Italy; Department of Urology, Federico II Naples University, Napoli, Italy

**Keywords:** Radical cystectomy, Ileal orthotopic neobladder, Quality of life, Specific questionnaire

## Abstract

**Background:**

The ileal orthotopic neobladder (IONB) is often used in patients undergoing radical cystectomy. The IONB allows to void avoiding the disadvantages of the external urinary diversion.

In IONB patients the quality of life (QoL) appears compromised by the need to urinate voluntarily. The patients need to wake up at night interrupting the sleep-wake rhythm with consequences on social and emotional life.

At present the QoL in IONB patients is evaluated by generic questionnaires. These are useful when IONB patients are compared with patients with different urinary diversions but they are less effective when only IONB patients are evaluated. To address this problem a specific questionnaire—the IONB-PRO—was developed.

**Methods:**

A) Based on a conceptual framework, narrative-based interviews were conducted on 35 IONB patients. A basic pool of 43 items was produced and organized throughout two clinical and four QoL dimensions. An additional 15 IONB patients were interviewed for face validity testing.

B) Psychometric testing was conducted on 145 IONB patients. Both classic test strategy and Rasch analysis were applied. Psychometric properties of the resulting scales were comparatively tested against other QoL-validated scales.

**Results:**

The IONB-PRO questionnaire includes two sections: one on the QoL and a second section on the capability of the patient to manage the IONB. For evaluation of the QoL, three versions were delivered: 1) a basic 23-item QoL version (3 domains 23-items; alpha 0.86÷ 9.69), 2) a short-form 12-item QoL scale (alpha = 0.947), and 3) a short-form 15-item Rasch QoL scale (alpha = 0.967). Correlations of the long version scales with the corresponding dimensions of the EORTC-QLQ C30 and the EORTC-BLM30 were significant. The short forms exhibited significant correlations with the global health dimension of the EORTC-QLQ and with the urinary subscales of the EORTC-BLM30. The effect size was approximately 1.00 between patients at the 1-year follow-up period and those with 3, 5, and > 5-year follow-up periods for all scales. No relevant differences were observed between the 12-item short-form and the Rasch scale.

**Conclusions:**

The IONB-PRO long and short-forms demonstrated a high level of internal consistency and reliability with an excellent discriminanting validity.

## Background

Muscle-invasive bladder cancer is the cause of 60-90% of all surgical bladder removals [1]. This neoplasia is still today a morbid condition with a high mortality rate. The objective of the surgical operation is to insure the best oncologic control of the disease at both local and systemic levels. Additionally the surgery aims to maintain urinary function over the medium and long-term associated with a satisfying quality of life (QoL).

The current surgical solutions are the ileal conduit as proposed by Bricker [2] and the ileal orthotopic neobladder (IONB). The former is an effective solution despite some aesthetic problems due to the urostomy [3]. Alternatively, the IONB entails a anastomosis between the intestinal neobladder and the urethra, allowing the patient to urinate normally [4,5]. Nevertheless, although the advantages of the IONB are evident, it is equally true that it can have a negative impact on the QoL of patients, such as the lack of bladder proprioceptive sensitivity obligating patients to volountarily void the reservoir every three hours even during the night. The wake-sleep rhythm of the patient is interrupted, thereby affecting daily life functioning and impacting on the patient’s social and emotional life if the patient fails to adapt to this condition.

In most of the studies conducted in the 1990s and in the beginning of the 2000s, the problem was comparing the IONB to other forms of urinary diversion (primarily the ileal conduit) [6-8].

Multi-domain generic questionnaires such as the MOS SF-36, or questionnaires generic by condition (cancer), such as the FACT-G (from FACIT) or the EORTC QLQ-C30 [9-11] have been used. In such study designs, generic instruments are the most suitable. They allow comparisons among different conditions, in exchange, however, for some degree of generality. Other study designs, such as cohort studies on IONB patients, or comparisons between IONB patient subgroups [12,13], could benefit relevantly from a more specific approach. Specific modules can therefore be attached to the generic questionnaire, but the burden on the patient is increased [14]. Shortcomings of such solutions motivated the authors of this manuscript to develop a new questionnaire—the IONB-PRO.

## Methods

Criteria for the IONB-PRO questionnaire are the following:

1. to be specific for monitoring patients with IONB over time;
2. to include two sections relative to: a) symptoms and patient IONB self-management (referred to as the IONB-S&M section); and b) QoL issues (referred to as the IONB-QoL section). The acronym IONB-PRO is used when referring to both sections, whereas the acronym IONB (alone) refers to the ileal orthotopic neobladder urinary diversion;
3. to be easy to administer having a maximum of 20–25 items overall with short-forms having 10–15 items;
4. to be reliable, having good construct validity and high discriminant capability among patients with IONB;
5. to have the potential to be developed into the primary European languages.

**Figure 1:**
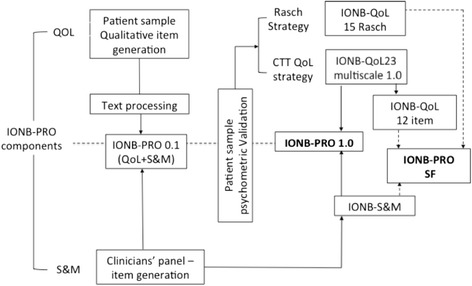
**IONB development roadmap.**

Methodological aspects were sketched on a roadmap (Figure 1) and summarized below

A.) Qualitative analysis methodology

**Figure 2:**
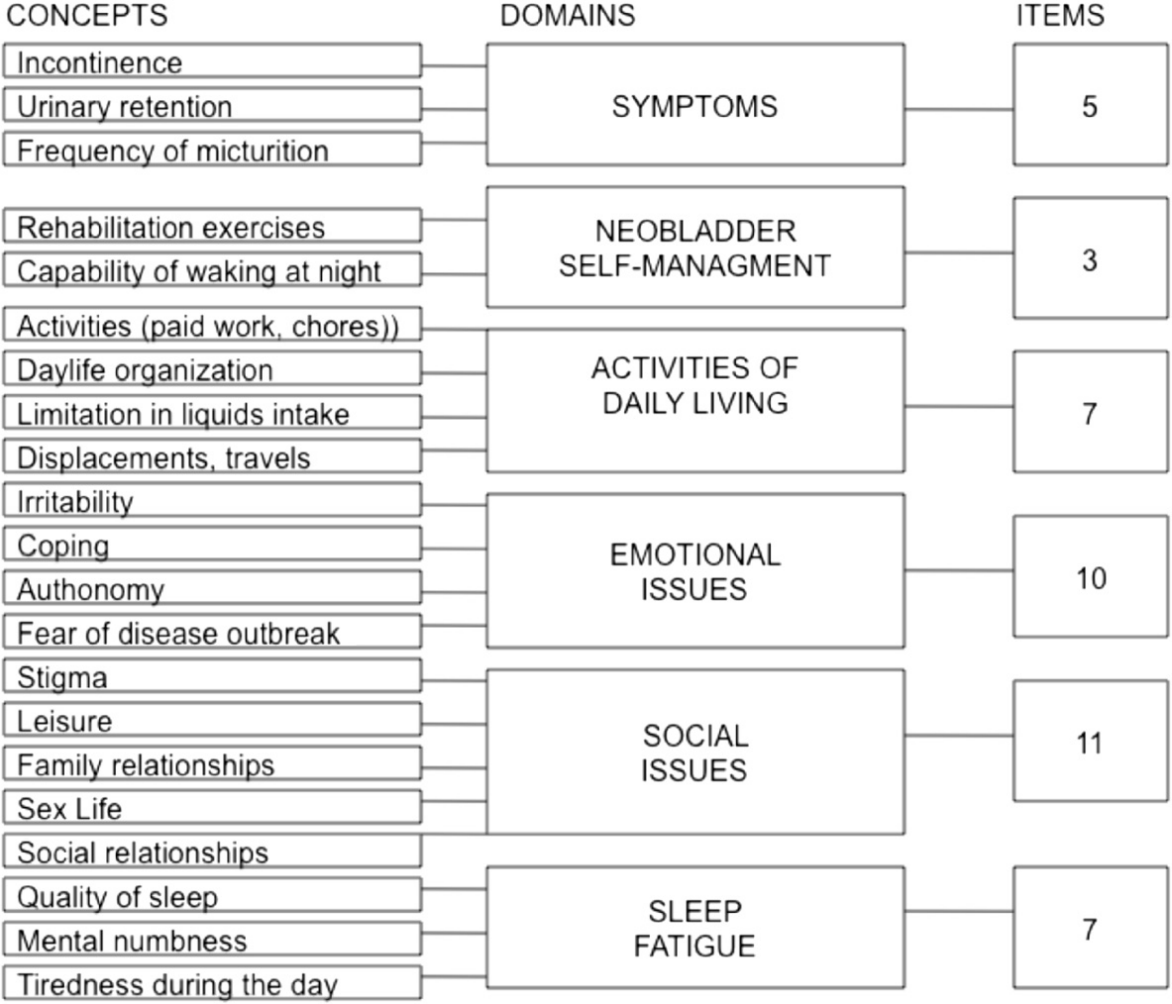
**Conceptual framework (Domains) with added concepts and number of items included in the IONB-PRO 0.1 for each domain.**

Production of a preliminary IONB-PRO 0.1 version. A preliminary hypothetical conceptual framework was drawn from the literature and/or discussed with a panel of clinicians (note blocks in the central column in Figure 2). Thirty-five patients from seven northern Italian Centres (Brescia, Bolzano, Trieste, Verona, Vicenza, Padova, and Modena) were recruited for narrative-based interviews [15,16]. The criteria for inclusion in the study were: having undergone a radical cystectomy for localized invasive bladder cancer or high-risk non-muscle-invasive bladder cancer and having received an IONB in the past year. Incontinent patients were separated into partially incontinent and hyper-continent patients. Approximately 40% of the patients were women. Interviews began with a generic question, thereby allowing the patient the freedom to speak about all issues he/she wanted. An interview guide with a list of probes according to the conceptual framework ensured that all important life events and experiences related to the disease were reported to the interviewer.

Scripts from the interviews were obtained and the text processing computer program Atlas.ti [17] was implemented in data analysis. The text from the interviews was coded into the main conceptually predefined categories (Figure 2).

A panel of clinicians generated a list of urinary symptoms and practical problems faced by patients living with a neobladder. This information was incorporated into the IONB-S&M section of the questionnaire.

Two formats of the questionnaire were prepared. One version with items expressed as statements, and a second version with items presented as questions. The response system included four categories (*always, often, sometimes, never*) with a time reference of "the last week."

The IONB-PRO 0.1 version so delivered was administered to 15 patients with an IONB. The style of inquiry was a cognitive interview [18] that consisted of asking the patient to complete the questionnaire by thinking aloud and by answering the questions of the interviewer (For example: *How would you formulate this concept in your own words? Is this instruction perfectly understandable? Why did you not complete this question?*).

B.) Quantitative analysis methodology

- Field testing general aspects
  The IONB-PRO 0.1 version was administered to a sample of 171 patients with an IONB from five University Clinics in Italy (Napoli, Padua, Trieste, Roman Catholic University, and Verona). This was a part of a more general study on survivors from radical cystectomy with either an IONB or with an ileal conduit. In the protocol, the following materials were included: the IONB-PRO 0.1 version; the EORTC-QLQ C30; the EORTC BLM30 module; and a Clinical Report Form (CRF) asking for a) demographics; b) clinical situation (follow-up months, pathological state, pathological lymphnodes, grading, incontinence, local situation of disease, metastasis, and ongoing chemo-radiotherapies); and c) co-morbidities.
  Patients were selected by starting with those in the charge of the Centre during one year since the surgical intervention, and proceeding backwards until sampling quotas assigned to each participating unit were met. Criteria for inclusion in the study included having undergone a radical cystectomy, having been treated with an IONB, being either males or females between 18 and 80 years old, capability of completing a questionnaire, being Italian speakers, and being exempt from cognitive deterioration. Conversely, criteria of exclusion included having psychiatric diseases, being substance addicts, and having difficulty in written and oral communication.
  For the purposes of psychometric property testing and item reduction, two different competing psychometric procedures were followed. The objective was that of producing questionnaires whose properties would be compared to determine the one exhibiting the best performance. The two competing reference theories are the Classical Test Theory (CTT) [19,20] and the Item-Response Theory (IRT), in particular, the Rasch model (Figure 1).

### IONB-QoL section: CTT analysis

The segment of the research applying the CTT or traditional Test Theory [21] started with exploratory Principal Component Analysis (PCA) [22] in order to confirm the multidimensional basic conceptual framework. This was followed by consistency analysis of the suggested scales by means of item-test correlations, the Cronbach alpha [23], and by dropping items that decreased reliability. A long-form three-dimensional IONB-QoL questionnaire was produced. Further item reduction occurred through step-wise regression by retaining a model suitable for minimizing the number of items and for maximizing the variance explained in comparison with the variance exhibited by the longer instruments. At each of these reduction steps, PCA was repeated [24,25]. A short-form IONB-QoL consisting of 12 items was produced.

### IONB- QoL section: Rasch analysis

The Rasch theory assumes that the condition of a person's health and the test capability to detect such a condition (we shall call “severity”) can be described on the same uni-dimensional (UD) linear logistic scale [24,26]. However, this basic assumption must be tested. The analysis aims to assess whether the data fitted the model. Misfitting items were rejected. In this research, the Rasch analysis was applied to the set of 35 IONB-QoL items by terminating the analysis when both the outfit and the infit of all items were within the suggested thresholds (0.5÷1.7) [27].

A 22-item scale resulted from the procedure, which, however, demonstrated disappointing performance. The analysis was subsequently repeated on a more restricted pool of items—those selected for the long scale IONB-QoL through the CTT, and those selected by using a tighter threshold range (0.5÷1.5) considered to be productive for measurement [28].

The initial Rasch analysis was checked through PCA of the standardized residuals [29,30] remaining from the application of the reduction of the previous item. Through a parallel analysis [31] the strength of the principal components was compared against the strength of the components generated by random noise. For this analysis, PCA was performed over 1000 matrices generated by random permutations of residual data. Each eigenvalue was compared with the respective 95^th^ percentile of random eigenvalues (λ_95_), and when it was greater than the respective λ_95_ value, the component was considered to be significant [32,33].

#### IONB-S&M section analysis

This section included items from two conceptual domains, relative to (a) "Urinary condition" including issues on the different types of urinary problems, and (b) "Capability of self-management of the IONB" including "capability of following the exercises suggested by the clinicians," "capability of emptying the IONB properly," and "waking at night."

For each o the two S&M domains Two-Step cluster analysis (distance measure log-likelihood; Schwartz’s Bayesian clustering criterion) was applied [21] and patients were divided into sub-groups according to the cumulative combination of critical issues.

#### Convergent, divergent, and discriminant validity

Spearman's Rho correlations were calculated by matching the IONB-QoL scales and subscales to the corresponding subscales of the concurrent measures included in the protocol. Values underlined in Table 1 represent hypotheses of convergence, of which several were found between the IONB-QoL (multidimensional version) subscales and those of the EORTC-QLQ C30 questionnaire. For the short-form QoL scales, no particular convergence was expected, except with the EORTC "Global Health" dimension.

**Table 1:** **IONB-QoL scale descriptives: convergent, divergent and discriminant analysis**

	**IONB-QoL 23 item**			**IONB-QOL 23 item 1D**	**IONB-QoL SF12 items**	**IONB-QoL 15 items (Rasch)**	**EORTC QLQ-C30**
	**Relational**	**Emotional**	**Fatigue**				
							**Global Healh**
*(A) Descriptives*							
Min	0	0	0	0	0	0	0
Max	100	100	100	98.6	97.2	100	100
Ceiling(%)	15.2	3.8	3.1	0.0	0.0	3.1	11.7
Floor(%)	16.5	10.7	13.8	7.6	8.9	8.7	2.3
Mean	58.42	51.101	43.606	53.165	51.055	54.065	64.549
SD	38.072	32.524	30.18	32.286	31.06	32.926	25.237
Cronbach StAlfa	0.969	0.943	0.859	0.974	0.947	0.967	0.918
SEM	6.703	7.765	11.333	5.206	7.151	5.981	7.227
*(B)* ^*1*^ *Concurrent measures (Rho) (Convergent/divergent validity)*							
QLQ-Physical Functioning	0.101	−0.043	**0.264	0.112	0.087	0.046	**0.636
QLQ-Role Functioning	*0.183	0.016	**0.280	*0.178	0.135	0.099	**0.643
QLQ-Emotional Functioning	0.036	*0.164	*0.199	0.131	*0.157	*0.163	**0.437
QLQ-Cognitive Functioning	−0.135	−0.039	0.091	−0.043	−0.021	−0.035	**0.337
QLQ-Social Functioning	0.007	−0.101	0.073	0	−0.034	−0.052	**0.583
QLQ-Fatigue	0.018	0.063	**-0.280	−0.037	−0.08	−0.034	**-0.554
QLQ-Sleep	−0.069	−0.112	*-0.161	−0.118	−0.112	−0.108	**-0.266
QLQ-Global Health	**0.235	0.138	**0.300	**0.250	**0.218	*0.199	1.00
BLM Increased frequency urin.	**-.603	**-0.658	**-0.454	**-0.642	**-0.651	**-0.676	0.122
BLM Urgency and loss of urine	*-0.278	*-0.191	**-0.295	**-0.293	**-0.272	**-0.251	**-0.505
BLM-Worry	−0.08	*-0.243	**-0.232	*-0.191	**-0.216	*-0.243	**-0.407
BLM-Body Image	0.034	−0.036	−0.045	−0.011	−0.009	0.003	**-0.380
*(C) Follow-up – Effect Size*							
1 year	0.00	0.00	0.00	0.00	0.00	0.00	0.00
3 years	1.09	0.97	0.45	1.02	0.98	1.08	0.14
5 years	1.57	1.41	0.76	1.49	1.47	1.64	−0.42
More	1.91	1.42	1.12	1.73	1.65	1.70	0.39
*(D) Urinary condition (means)*							
Incontinent	62.709	56.293	39.286	56.403	54.308	59.863	46.959
Regular	88.384	72.264	62.438	78.107	74.242	77.946	74.552
Incontinent/hypercontint	7.541	12.209	19.186	11.19	11.757	10.284	69.279
*(E) IONB management*							
Continent	84.447	71.201	59.559	75.512	71.446	75.556	73.588
Difficulty compress/decompress	64.141	56.684	43.056	57.88	56.424	61.064	48.979
All problems	9.74	13.081	18.992	11.594	11.905	10.740	67.906

*Correlation is significan at the 0.05 level (2-tailed).

**Correlation is significant at the 0.01 level (2-tailed).

The EORTC-QoL subscales and the short forms were expected to correlate with the dimensions relative to "urinary symptoms" and "worry" of the specific EORTC-BLM30 module. Divergence was expected between all IONB-QoL subscales and the short forms with the QLQ C30 "Cognitive functioning" and the BLM30 "Body Image" categories.

Validity was considered to be convergent if Rho was significant at p < 0.05. In contrast, correlations were specified as divergent when they were non-significant [19].

Discriminant validity [34] was tested by checking the capability of the scales to distinguish among patients grouped by three critical variables: 1) the follow-up period—the effect size (ES) was calculated between patients with a 1-year follow-up period and those with 3, 5, and > 5-year follow-up periods—the underlying rationale being that the QoL is expected to worsen abruptly after surgical intervention, while improving as the patient adapts to the condition; 2) clustering patients by "urinary" condition (from the IONB-S&M)—the rationale being that belonging to either cluster would affect a change in the QoL; and 3) clustering patients by IONB self-management capability (from the IONB S&M section)—by the same rationale as stated in point 2.

Because most scale distributions were determined to be non-normal, the nonparametric Kruskal-Wallis test was applied for discriminant validity analysis; the Mann–Whitney nonparametric test was then applied for post-hoc comparisons, each with Bonferroni correction of alpha in order to maintain the overall probability of a type I error at 0.05.

The computer programs used throughout this study included: Atlas.ti for qualitative analysis of the narrative-based medicine interviews [35]; Winsteps 3.80.0 for Rasch analysis [25]; and R and SPSS version 17 for other calculations [21,36].

The study was conducted with approval of the Ethics Committee appointed for each Centre, and alla patients signed and informed consent form.

## Results

### Qualitative analysis

The primary demographics of the patient sample used for qualitative analysis included 28 males, 7 females, with an average age of 63.3 years, of whom 21 patients were continent, 10 patients were incontinent only at night, 2 patients were totally incontinent, and 2 patients were hyper-continent. Each interview lasted between 45 and 90 minutes.

Patients were divided into two main groups according to the content of their responses—patients with good adaptation to the new condition or patients lamenting poor or critical adaptation to living with the IONB. Those who were well adapted tended to have at least two issues in common: firstly, successful rehabilitation, and secondly, most of them felt the stimulus to urinate, which allowed them to be in better control of micturition by avoiding embarrassing situations. Although patients lamented problems in their sex life, such patients also had in common a younger age, a supportive family, and a network of friends. More or less all of them related that their lives had become similar to that before the surgical intervention. Conversely, living with the neobladder was difficult for the poorly adapted group. The most relevant concern was their incapability of controlling incontinence, and the necessity of depending on others, both currently as well as in the future. Their lives constantly revolve around the fears of odors, the need to always be close to a toilet—which is not possible in several circumstances—and micturition, which gives rise to a complicated organization of life, especially for those who are hyper-continent and must catheterize several times a day. Patients of the former group seem to have developed successful coping mechanisms, while those of the latter group have not. For these patients, it is more difficult to wake at night while feeling numb and sleepy during the day; they must carry in a bag all necessary items (pads in the case of partial incontinence or catheters in the case of hyper-continence). They need to wear pads during the night-time, and they find it difficult to meet prescriptions, especially in regard to fluid intake. Problems having sex affected both groups but are felt in a more dramatic way by the latter group of patients, some of whom avoided sex and sexual situations, especially if they had no fixed partner. Poor adaptation to the condition is concomitant with a dramatic psychological profile (irritability, insecurity), in addition to feeling physical fatigue during the day as a consequence of sleeping badly at night.

A summary of the concepts emerging from interviews is presented in the left-hand column of Figure 2. In the central column of Figure 2, the basic domains are listed, while in the right-hand column the number of items retained for each domain, are presented. The basic pool comprises 43 items used for subsequent scale development, which are reported in Table 2.

**Table 2:** **Map of items by scales in IONB-PRO development**

**Number**	**Item**	23item**	**12item**	**15Rasch**
IONB1	Incontinence daytime			
IONB2	Incontinence nightime			
IONB3	Regular micturition			
IONB4	Feverish sensation			
IONB5	Difficult urinating			
IONB6	Difficulty compressing-decompressing IOB			
IONB7	Emptying bladder			
IONB8	Bothering waking at night			
IONB9	Difficulty in light physical activity			
IONB10	Fear of being far from toilet			
IONB11	Limited in drinking liquids			
IONB12	Able to organize time			
IONB13	(Avoided public transports)			
IONB14	(Less productive on work)			
IONB15	Limited activities	3		
IONB16	Feeling independent	1		
IONB17	Living well with neobladder	1		
IONB18	Felt angry	2	x	
IONB19	Felt panicking	2		X
IONB20	Felt irritable	2	x	X
IONB21	Felt hopeless	2	x	X
IONB22	Fear that the disease went on	2	x	X
IONB23	Worries for the future	2		X
IONB24	Felt diminished	2		X
IONB25	Losing self-esteem	2		X
IONB26	Slept bad at night			
IONB27	Felt tired daytime	3	x	X
IONB28	Waking refreshed in the morning			
IONB29	Needed to rest daytime	3	x	
IONB30	Get tired easily	3	x	X
IONB31	Less bright in doing things			
IONB32	Had to stop doing because of tiredness			
IONB33	Support from families			
IONB34	Difficult to get on with people	1		
IONB35	Avoided leasure activities	1	x	
IONB36	Avoided to go out	1	x	X
IONB37	Avoided to stay close to people	1	x	X
IONB38	Felt embarrassed	1		X
IONB39	Felt different	1	x	X
IONB40	Fear in meeting new people	1		X
IONB41	Afraid of being rejected from friends	1	x	
IONB42	Avoided physical contacts	1		X
IONB43	Avoided situations of physical intimacy			

(*)Items are written in order of appearance in the protocol questionnaire IONB-PRO 0.1. Domains are underlined in grey: “Urinary situation” (IONB1-5), “Bladder management” (IONB6-8), “Activities of daily living” (IONB9-15), “Emotional issues”(IONB16-25), “sleep and fatigue”(IONB26-32), “Social issues”(IONB33-43). (**)In the 23 item column, number 1 stands for “Relational life”, 2 for “Emotional life”, 3 for “Fatigue”.

Fifteen patients underwent cognitive interviewing for face validity testing after the administration of the IONB-PRO 0.1 version of the questionnaire. Completion of the questionnaire required an average of 15 minutes for 34 questions. Specific issues noted were: 1) item formulation—patients preferred the version with the items formulated as questions instead of statements; 2) phrasing—all items were perfectly understandable; 3) some of the patients argued that some of the items were not applicable for many, such as "less productive at work" or "avoided public means of transport” ; 4) response system—the distribution of answers were perceived to be concentrated on the upper part of the response options (i.e., never). Nevertheless, no suggestions were made for dealing with these problems.

### Psychometric validation

Demographic and clinical descriptives for the 171-patient psychometric sample are given in Table 3.

**Table 3:** **Clinical descriptives**

**Follow-up**	**%**	**Grading**	**%**	**Pathological state**	**%**	**Pathological lymphonodes**	**%**
1 year	16.4	G1	1.2	T1	31.5	Yes	2.4
3 years	33.3	G2	10.7	T2	47.6		
5 years	12.9	G3	86.3	T3	18.5	Gender	%
More	37.4	G4	1,8	T4	2.4	F	8.8
Disease onset	%	Metastasis	%	Age		Percentiles	Age
Yes	5.3	Yes	1.8	Mean	64.33	25	57.00
				St.dev	9,377	50	66.00
						75	74,00

### IONB-S&M sections

For the dimension "Urinary condition," cluster analysis suggested the following groupings: cluster 1 = incontinent (n = 49); cluster 2 = continent(n = 67); cluster 3 = incontinent & hypercontinent (n = 43).

This typology was compared against all other items and subscales throughout the protocol. Cross tabulation between an item of the CRF—"loss of Urine"—demonstrated a Chi Square value of p < 0.000. The Analysis of Variance (ANOVA) between the EORTC-BLM and items 1–7 (made more efficient if divided according to the PCA into two sub-dimensions—"urinary frequency" and "urgency") showed Fisher F values of p < 0.000 and p = 0.001, respectively.

From the items of the dimension "Patient's IONB self-management" the following clusters were produced: cluster 1 = continent (n = 68); cluster 2 = difficulty compressing/decompressing bladder (n = 48); cluster 3 = all problems (those of cluster 2 and difficulty waking-up at night) (n = 43).

Unfortunately, little information exists regarding possible concurrent measures for this latter typology. Therefore, further validation of this dimension will be left to future research.

### IONB-QoL sections

**Figure 3:**
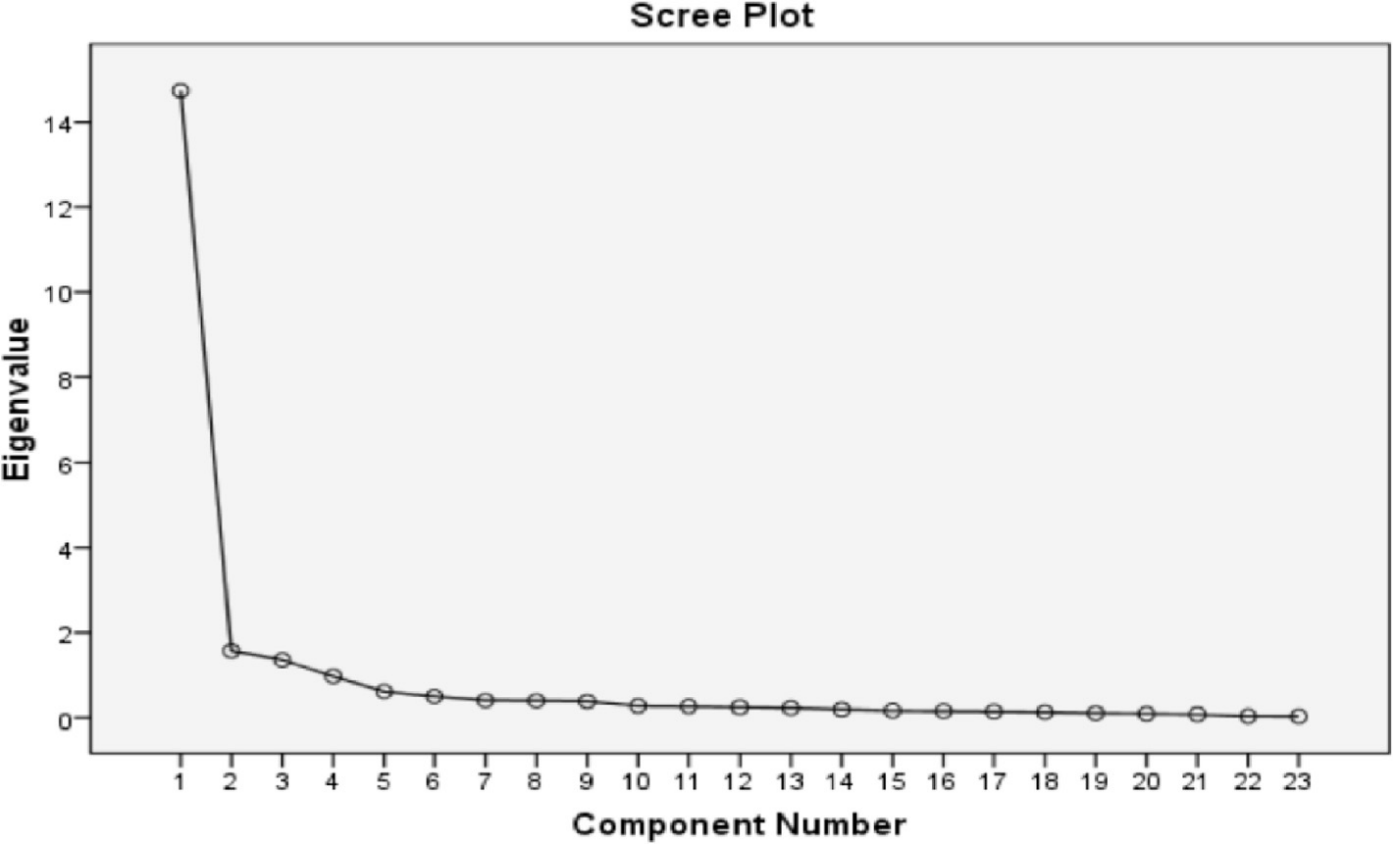
**Scree plot basic IONB-QOL basic version.**

Preliminary exploratory PCA applied to the 33 items of the IONB-QoL questionnaire extracted 5 (varimax-rotated) factors explaining as much as 76.918% of the overall variance. Four of the factors were related to highly saturated items: 1) "social issues" (16 items); 2) "activities of daily living" (7 items); 3) "emotional issues" (6 items); and 4) "tiredness and fatigue" (5 items), while the fifth factor included the single item 5) "I wake refreshed in the morning." While there were 5 components with eigenvalues greater than 1, the scree plot suggested an underlying one-factor structure. Each of the scales underwent reliability analysis in order to optimize internal consistency. As a result, 12 items were dropped. A PCA repeated on the remaining 23 items exhibited 3 components explaining 76.818% of the variance. The dimension "Activities of daily living" collapsed into the dimension "sleep and fatigue," and the new dimension was labeled FATIGUE (4 items with StAlpha (Standardized Alpha) = 0.855). The scale on "social issues" was renamed RELATIONAL LIFE by including 10 items with StAlpha = 0.969. Emotional issues were renamed EMOTIONAL LIFE and included 7 items with StAlpha = 0.944. The scree plot is illustrated in Figure 3 and demonstrates that a UD solution could have been acceptable. These 23 items were determined to represent the basic three-dimensional IONB-QoL questionnaire. Scaled scores were transformed into scales ranging from 0–100, in which 0 was the worst condition possible, and 100 was the best condition.

Step-wise regression was applied to the 23 items that were collapsed into a UD scale score, and they were included into the regression model as a dependent variable with the individual items as predictors. Results are reported in Table 4 showing that by selecting a regression model with 12 items the loss in variance explained was less than 1% of that explained by the original 23-item scale.

**Table 4:** **Step-wise Regression on the IONB-23 uniscale: Model summary**

**Model**	**R**	**R2**	**Adj R2**	**Std. Error**	**Variables in the model**
1	.921	0.848	0.847	12.647	a. Predictors: (Constant). Avoided to stay close to people
2	.958	0.918	0.916	9.330	b. Predictors: (Constant). previous + Felt hopeless
3	.972	0.944	0.943	7.699	c. Predictors: (Constant). previous + Avoided leasure activities
4	.979	0.958	0.957	6.689	d. Predictors: (Constant). previous + Afraid of being rejected from friends
5	.984	0.969	0.968	5.811	e. Predictors: (Constant). previous + Get tired easily
6	.988	0.977	0.976	4.981	f. Predictors: (Constant). previous + Fear that the disease went on
7	.992	0.984	0.983	4.152	g. Predictors: (Constant). previous + Feeling Independent_r
8	.994	0.987	0.987	3.732	h. Predictors: (Constant). previous + Feeling angry
9	.995	0.990	0.990	3.284	i. Predictors: (Constant).presious + Avoided to go out
10	.996	0.992	0.991	3.023	j. Predictors: (Constant).previous + Felt tired daytime
11	.996	0.993	0.992	2.807	k. Predictors: (Constant). previous + Feeling panick
12	.997	0.994	0.993	2.630	l. Predictors: (Constant). Previous + Needed to rest daytime
13	.997	0.995	0.994	2.463	m.Predictors: (Constant). Previous + Felt different
14	.998	0.996	0.995	2.258	n. Predictors: (Constant). previous + Felt irritable
15	.998	0.996	0.996	2.093	o. Predictors: (Constant), previous + Losing self-esteem
16	.998	0.997	0.997	1.892	p. Predictors: (Constant). previous + Living with neobladder_r
17	.999	0.997	0.997	1.737	q. Predictors: (Constant). previous + Difficult to get on with people
18	.999	0.998	0.998	1.528	r. Predictors: (Constant). previous + Worries for the future
19	999	0.999	0.998	1.293	s. Predictors: (Constant).previous + Limited activities
20	1.000	0.999	0.999	1.027	t. Predictors: (Constant). previous + Avoided physical contacts
21	1.000	1.000	1.000	0.676	u. Predictors: (Constant). previous + Felt handicapped
22	1.000	1.000	1.000	0.459	v. Predictors: (Constant). previous + Felt embarrassed
23	1.000	1.000	1.000	0.000	w. Predictors: (Constant), previous + Fear in meeting new people

Disappointing results from the initial Rasch analysis initiated additional analyses of the pool of 23 items selected for the IONB-PRO questionnaire. The procedure identified 15 items with the outfit included between 0.43 and 1.46 and the infit included between 0.56 and 1.30, while eight items were dropped. Table 5 shows the items removed at each step as well as the respective fit indices.

**Table 5:** **Items showing misfit that were deleted**

	**Infit**	**Outfit**	***R***
IONB18	5.60	9.00	−0.63
IONB34	1.02	1.92	0.76
IONB16	1.34	1.70	0.72
IONB15	1.36	1.81	0.67
IONB17	1.70	2.01	0.71
IONB29	1.57	1.82	0.60
IONB35	1.40	1.61	0.71
IONB41	0.52	0.36	0.86

The severity associated with the items of the reduced questionnaire were plotted against the distribution of individual's conditions (figure not reported). The measurements of the items varied between −0.44 and 0.56 logits with thresholds included between −2.47 and 2.24 logits. Consideration of the individual's conditions varied between −5.26 and 5.08 logits, thereby being demonstrated to be far from the assumptions of the Rasch model.

The infit values indicated that 27.9% of individuals misfitted the model (overfit: 12.4%; underfit: 15.5%), and this percentage increased to 34.2% when the outfit analysis was added (overfit: 20.50%; underfit: 13.66%).

As far as the unidimensionality of the scale is concerned, the PCA on standardized residuals found one component with an eigenvalue greater than 2 (λ = 3.3). Parallel analysis found this value to be greater than the expected λ_95_ value obtained by random permutations of residual values (λ_95_ = 1.7). Nevertheless only 1.9% of the Pearson's correlation between residuals exceeded the values −0.4 and 0.4, indicating the weakness of correlations between residual components. The total unexplained variance was 33.9%, and the residual component greater than 2 accounted for 7.5% of the total variance. A summary of the items in the scale is provided in Table 6.

**Table 6:** **Result of Rasch analysis by measure (location): Infit and Outfit MSQ values**

**Item**		**Mea-sure**	**SE**	**Infit MSQ**	**Outfit MSQ**
IONB37	Avoided close to people	−0,44	0.13	0.56	0.51
IONB40	Fear meeting people	−0,42	0.13	0.61	0.43
IONB38	Embarrassed	−0,40	0.12	0.72	0.63
IONB36	Avoid to go out	−0,25	0.12	0.91	0.98
IONB42	Avoid physical contacts	−0,13	0.12	1.06	0.83
IONB39	Felt different	−0,10	0.12	0.76	0.79
IONB25	Losing self-esteem	−0,05	0.12	0.61	0.59
IONB19	Panicking	−0,04	0.12	0.91	0.85
IONB21	Hopeless	0,01	0.12	0.73	0.65
IONB20	Irritable	0,04	0.12	1.18	1.17
IONB23	Worries for the future	0,10	0.13	1.24	1.35
IONB22	Fear disease goes on	0,22	0.13	1.30	1.41
IONB24	Felt diminished	0,43	0.12	1.15	1.12
IONB27	Felt tired daytime	0,47	0.13	1.30	1.40
IONB30	Get tired easily	0,56	0.12	1.28	1.46

### Construct validity of the IONB-QoL sections and scales

A map of the various scales and their corresponding items after the operations described above is presented in Table 2. The descriptives of such QoL scales are given in Table 1 including, among others, the percentage of cases at the ceiling and at the floor; the internal consistency reliability Cronbach StAlpha and the standard error of measurement (SEM) as an estimate of the minimal clinical important difference (MCID) [37,38].

Table 1 shows Spearman's correlation coefficients for the EORTC measures. Strong convergence was observed (with high correlations) between all scales and specific concerns such as "worry for the future" (EORTC-BLM30). As expected, the subscales of the IONB-QoL 23, showed pertinent convergence with the corresponding scales of the EORTC-QLQ. Instead, the IONB-QoL short form scale correlations shifted from those with the domains of the EORTC measures to the "global health" dimension of the same QLQ C30 questionnaire.

Discriminanting validity between follow-up groups was analyzed through the ES [37,39] and was calculated between the follow-up at year-1 and the other follow-up patient groups (mean deltas divided by the standard deviation of the group at year-1). The results are given in Table 1C, recalling that Cohen [37] interpreted ES as small when < 0.5, medium when 0.5 ÷ 0.8 and large when > 0.8. The means of all subscales and short forms of the IONB-QoL questionnaire obtained by clustering of the IONB-S&M dimensions are shown in Tables 1D and E. For both, the Kruskal-Wallis nonparametric test was applied, showing p < 0.000, while the Mann-Withney nonparapetric test (Bonferroni correction) was applied with post-hoc analysis. All multiple comparisons for each set of clusters were significant at p < 0.000, with the exclusion of the comparison between "continent" patients and "incontinent and hypercontinent" patients for the clusters on "urinary condition" which were non significant. The same observation existed between "continent" patients and those reporting "having all problems" of the clusters on "IONB-self management."

In order to obtain a possible reference value for discriminant validity, the same measures were calculated for the EORTC domain "Global Health," whose performances are compared to those of the IONB-QoL scales and subscales in this study, and are reported in Table 1 A-D.

## Discussion

Three positive issues of the IONB-QoL scales were observed. First is their internal consistency. Inter-item correlations of the subscales of the multidimensional version 1.0 as well as that of the short-form scales rarely were below r = 0.6, while the Cronbach alpha was always greater than 0.9 (an exception was made for the sub-dimension "fatigue" where alpha = 0.859). Several other psychometric properties benefited from the high degree of internal consistency. One of these was the SEM from which the estimate of the MCID was derived (Table 1A) [35,37] that makes score changes more clinically interpretable. Another advantage was demonstrated by the step-wise regression in which the level of variance explained by the various regression models was high even after a relevant number of items were dropped (Table 4).

A second positive aspect was demonstrated in Table 4. Among the models selected by the step-wise regression procedure, even those with a small number of predictors included items belonging to all of the components of the initial conceptual framework (Relational, Emotional, Activity, and Fatigue). Good stability of content validity was observed even in the extreme case in which only 7–8 items were retained (note in that case the loss of variance explained in comparison to the initial 23-item model was only 1%). Although a lower threshold could have been used, examining the short-form scale was terminated at 12 items. A third positive aspect determined was the excellent discriminant ability of any of the scales toward all of the sub-groups of patients that were tested—by follow-up period, by urinary condition, and by capability of IONB self-management.

The specific properties of the IONB-QoL (long and short form) sections can be appreciated especially if compared with those obtained from the otherwise excellent generic measure "Global Health" (domain of the EORTC QLQ-C30). Figures provided by the latter measure do not show any apparent order if applied to the same groups of patients (note the last columns of Table 1C, D, and E) and this best demonstrates the specific nature of the IONB-PRO questionnaire. In addition, the scale “Global Health” failed to distinguish significantly between patients belonging to crucial clusters relating to urinary problems and IONB self-management capability.

In addition to such positive aspects, negative issues were also observed. One of these is the problem of the uni-dimensionality of the scales. The literature demonstrates a variety of methods for factor extraction [40]. Three of these have been applied in this research, each producing different results. The method of selecting the eigenvalues greater than 1 [22] suggests a three- or four-factor solution. Cattel’s method of generating the scree plot [41] suggested a UD scale instead. The method of the PCA on the standardized residuals, suggested by the authors of the Rasch approach [29,30,32,33], showed some "signals" of multidimensionality although the strength of the residual components and their proportion of explained variance appear negligible.

Aware of such contradictions, sensitive choices were made in this research by first working on the multiple scale hypothesis and by subsequently diverting to the hypothesis of unidimensionality as long as the number of items decreased. The efficiency shown by the scales applied and the fact that they adhere to the basic concepts of the study are encouraging.

Other relevant shortcomings are the floor and ceiling effects of the IONB-QoL scales and subscales (Table 1A). Such effects are both relevant in subscale RELATIONAL: 16.5 and 15.2%, respectively. In all other scales the ceiling effect tends to fade; however, the floor effect never was observed to be less than 7%. This could be due to the tendency of responses on QoL issues to concentrate at the extremes in a U-shaped distribution. This separation between patients that were adapted to living with IONB and those who were not was evident since the beginning of the qualitative interviews and could be a constitutional feature of the distributions. Data showed that this division affected all of the IONB-QoL scales, but it was particularly evident in the scale RELATIONAL. Note for example that its standard deviation is higher than that of the other distributions in Table 1A. All of the standard deviations of the IONB-QoL subscales and short forms are greater than that of the EORTC subscale "Global Health," which showed a nearly normal distribution. All EORTC scales as well as the IONB-QoL were transformed on the same 0–100 scale.

Other considerations, however, lead to the hypotheses that the items and/or response scales are biased. An indication of a problem was observed in the Rasch analysis, which showed good reliability with person “condition” but reduced reliability with the item “severity” primarily due to a narrow variance on the Rasch analysis for the parameter. Specifically, the distribution of measures for person extends beyond those for items, making it difficult to distinguish between extreme outcomes. The tendency of patients to use the extremes of the response scales were also observed during the cognitive interviews. The solution in this case could be to reduce the scale steps by configuring the following: *always* (as is), *often* (instead of *sometimes*), *sometimes* (instead of *rarely*), *never* (as is). This alteration should balance distributions by channelling patient answers toward the central items of the response scale.

A final question is whether conducting Rasch analysis as documented in this article was worth the effort. Actually we undertook this study in the conviction that the Rasch procedures would be the most effective in questionnaire development. Examination of the results of calculations made according to the traditional psychometric procedure provided evidence that the resulting scales were more applicable. The typical violation of one of the Rasch model preconditions, namely the relevant discrepancy between the patient's condition and ability of the item to describe it (severity), provides evidence that the items used were not suitable for the Rasch analysis. Expressly, this is a case in which researchers will have to admit that the CTT was more effective. Conversely, the observations in Table 6 in which the fitted items are ordered by "measure" (or weight, location, importance) indicated that the output of the Rasch procedure offered more insight to the data. Items on RELATIONAL issues are those that were observed to be more severe in comparison to the others. The problems they describe affect a relatively small number of patients, while items on FATIGUE are more widespread and represent a less severe condition, typical of all patients with IONB. EMOTIONAL items were located in the middle, and these are the items for which the distributions were tendentially normally shaped, indicating that RELATIONAL aspects (including stigma, meeting new people, etc.) were those that can most contribute to making the quality of life unbearable.

## Conclusions

This research leaves some questions unanswered. Future research should address other study designs; whether the short 12-item or 15-item versions should be retained; the improvements to be gained by applying a different response scale; and, whether the U-shape of some of the distributions is a bias or a feature that reflects the actual patient condition. Meanwhile, researchers using the IONB-PRO questionnaire should use nonparametric statistical tests.

The results obtained to date are very interesting. The IONB-PRO questionnaire, in all long and short forms, demonstrated good face and content validity, a high level of internal consistency and reliability, acceptable construct validity, and excellent discriminant validity. All project requirements were met, including that of producing a very specific instrument less than 20 items long.
